# Developmental Delay and Macrocephaly Unraveling a Leukodystrophy: A Case Report

**DOI:** 10.7759/cureus.105905

**Published:** 2026-03-26

**Authors:** Monica Potru, Ankita Pandey, Jitesh Rawat, Purnendu Shekhar, Kanika Khandelwal

**Affiliations:** 1 Department of Radiology, Dr. Rajendra Gode Medical College, Amravati, IND; 2 Department of Radiology, Jawaharlal Nehru Medical College, Aligarh, IND; 3 Department of Internal Medicine, Mayo Clinic Health System, Austin, USA

**Keywords:** delayed motor milestones, macrocephaly, megalencephalic leukoencephalopathy, mlc1 gene mutation, pediatric neurology, subcortical cysts, van der knaap disease

## Abstract

An uncommon inherited autosomal recessive disorder, Van der Knaap disease is also referred to as megalencephalic leukoencephalopathy with subcortical cysts (MLC). Megalencephaly, which either develops at birth or during infancy, along with seizures and a mild motor development delay, is its defining characteristic. Leukodystrophy and subcortical cystic degeneration are the two MRI abnormalities that are indicative of the illness and typically provide the key to diagnosis. The disease is more common in certain ethnicities, like the Aggarwal community in India, where marriages within the community are common. Here, we describe an Indian patient from a non-Aggarwal community born out of a non-consanguineous marriage who had Van der Knaap disease with the typical MRI features.

## Introduction

Van der Knaap disease is a form of slowly progressive hereditary white matter neurodegenerative disease. Clinically, the disease is usually presented at birth or in early infancy by macrocephaly with normal or mildly delayed developmental milestones. Later, the disease may advance to gait abnormality and seizures, and a few cases show dementia [1]. In the majority of cases, mutation is seen in the MLC1 gene located on chromosome 22q [1,2]. In some ethnic groups where consanguinity is prevalent, such as the Aggarwal community in India, it happens more frequently, and thus, the condition is sometimes also called Aggarwal’s disease [3]. The classical findings seen in brain MRI are diffusely swollen white matter and subcortical cysts. Gradually, cerebral atrophy is seen in the later course of disease [1]. We present a case of a four-year-old boy from western Uttar Pradesh from a non-consanguineous Hindu marriage from a non-Aggarwal community who presented in the pediatric neurology outpatient department with complaints of difficulty in walking, and on further discussion, the parents mentioned that they feel his head has been progressively increasing in size since birth, though macrocephaly was not identified in any antenatal scans. The patient was having mildly delayed developmental motor milestones. Based on his brain MRI classical traits, he was diagnosed with megalencephalic leukoencephalopathy with subcortical cysts (MLC).

## Case presentation

A four-year-old boy from a non-consanguineous Hindu marriage from the northern Indian state of Uttar Pradesh came to our pediatric neurology outpatient program complaining of walking difficulty since he was a young toddler. Neck holding came up at eight months of age, and the child started walking by 1.5 years of age, representing a delay in motor developmental milestones.

He was delivered vaginally at full term with the birth weight being low, i.e., 1.25 kg, and a history of delayed cry for 2-3 minutes after birth. A year ago, the boy experienced seizure episodes, which the parents also complained about. Blurred vision, head injuries, or a protracted fever were not present in the past. There was no significance to family history.

**Figure 1:**
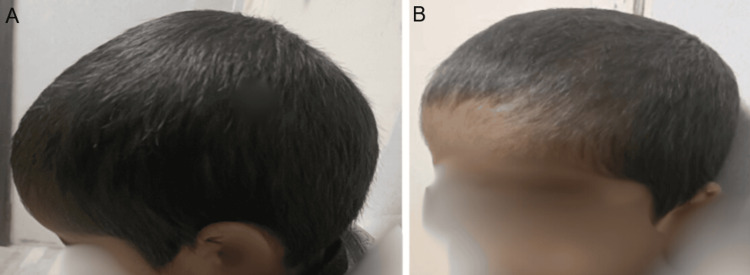
(A, B) A 4-year-old child with macrocephaly

**Figure 2:**
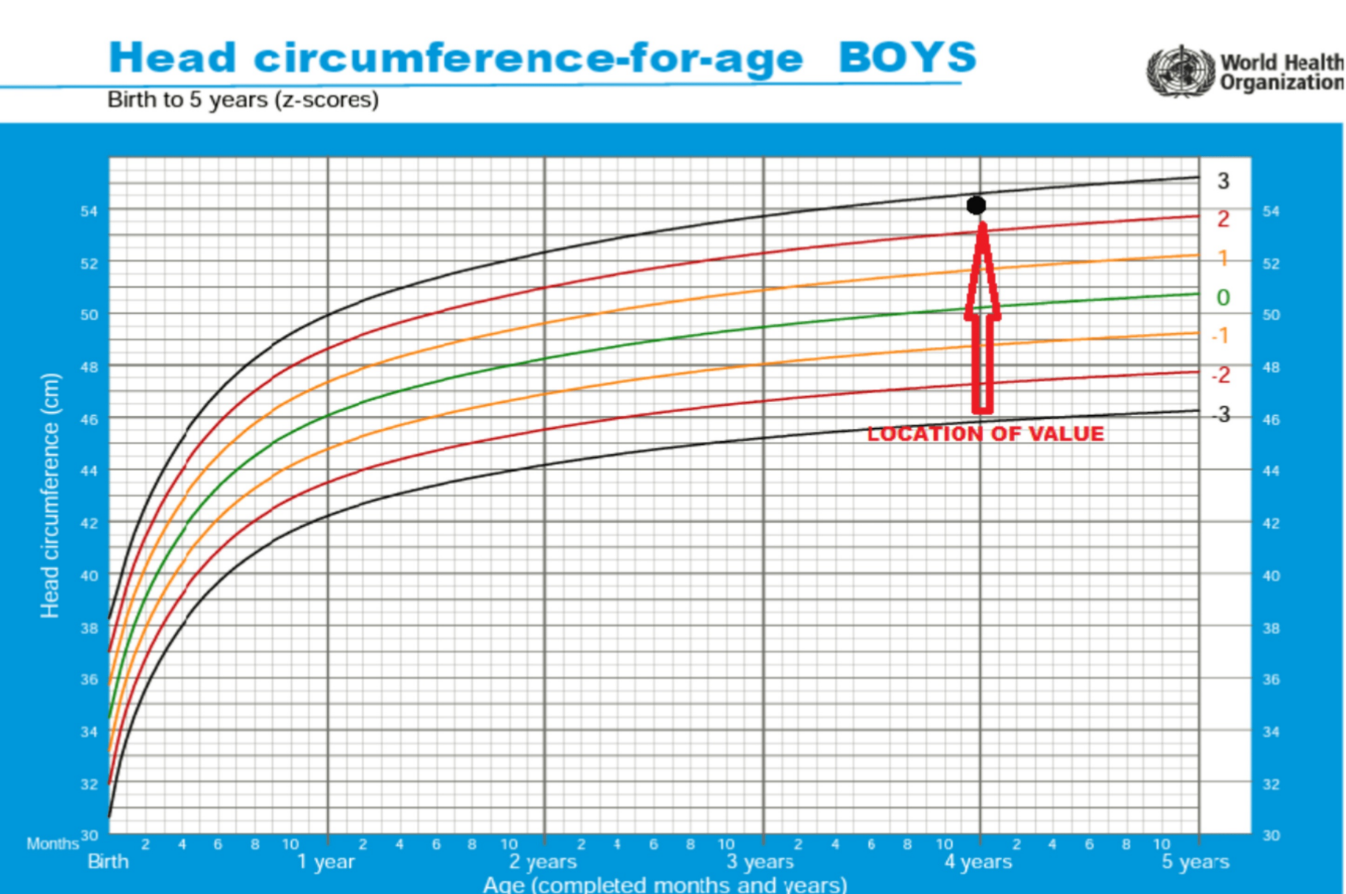
Child’s head circumference lies above the 95th percentile in the WHO growth chart, showing head circumference-for-age in boys Source: Reference [4]. Image modified by the authors

On examination, the baby's head circumference measured 54 cm, which was greater than the 95th percentile for his age, indicating macrocephaly (Figures 1, 2). The patient was having toe gait while walking, and the power in both lower limbs was 3/5. The remainder of the systemic and general physical examination was uneventful.

Investigations

**Figure 3:**
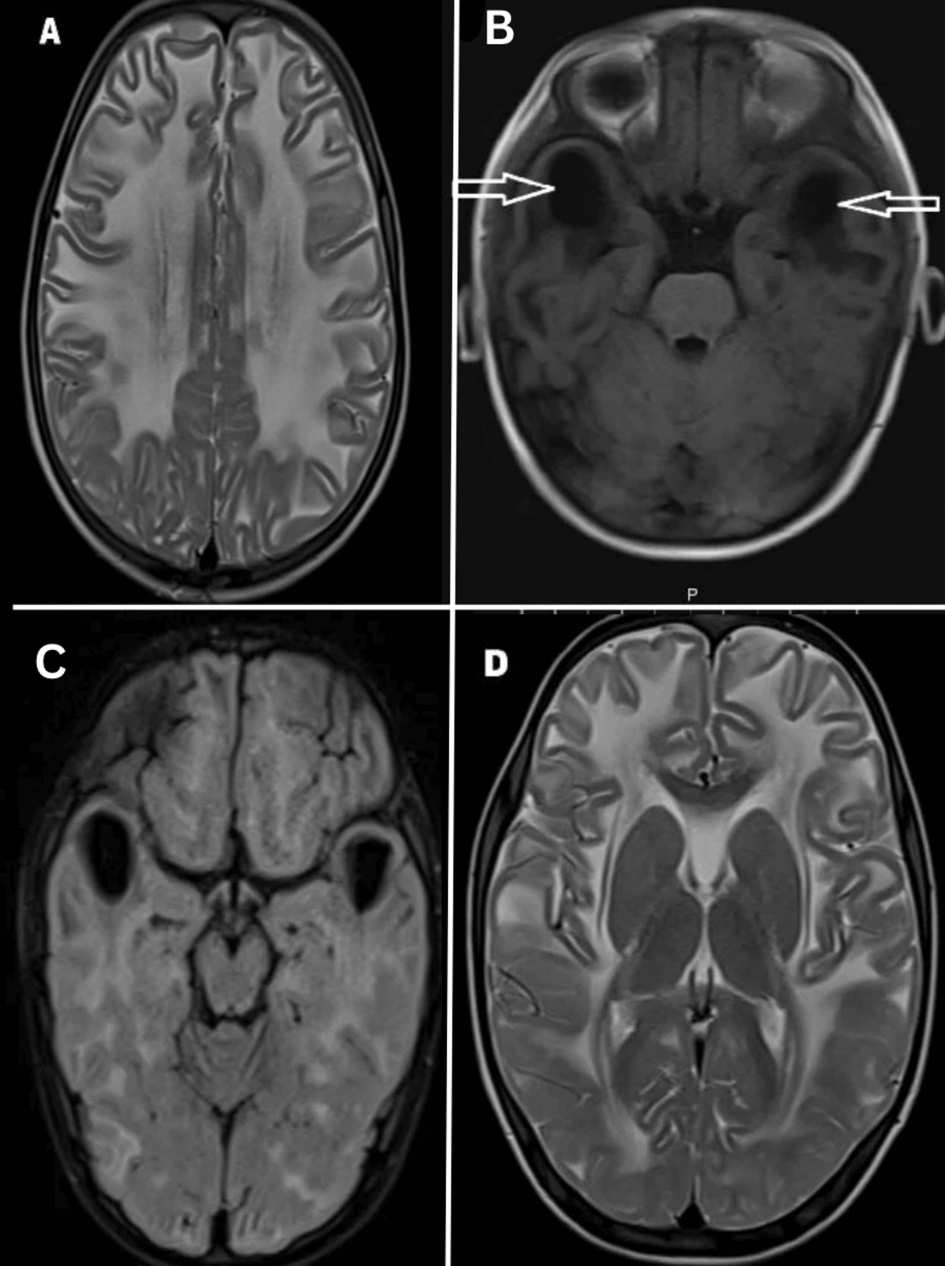
Brain MRI T2 WI (A) shows symmetrical diffuse bilateral hyperintensity (dysmyelination) involving subcortical and deep white matter of cerebral hemispheres along with formation of subcortical cysts bilaterally in the frontotemporal lobe as seen in T1 WI (B) and T2 fluid-attenuated inversion recovery (FLAIR) (C) sequence. B/L basal ganglia and B/L thalamus are spared as seen in T2 WI (D) WI: weighted imaging

Routine blood investigation tests, chest X-rays, and abdominal ultrasounds were normal. Brain MRI showed diffuse bilateral symmetrical hyperintensity on T2-weighted imaging and hypointensity on T1-weighted imaging involving the white matter of the cerebral hemispheres with a swollen appearance, with involvement of subcortical U fibers as well. There was a subcortical cyst formation with CSF intensity involving the B/L frontal and temporal regions (Figure 3). Septum pellucidum vergae was noted (not shown here). There was no evidence of any restriction on diffusion-weighted imaging.

CSF analysis was not done. In view of typical MRI imaging findings and history of macrocephaly with delayed motor milestones, we kept Van der Knaap disease as the diagnosis.

Differential diagnosis

Alexander disease, Canavan disease, and a few biochemical disorders are the common differential diagnoses of Van der Knaap disease, but these conditions will have a relatively quick progression of disease and a more clinically evident presentation, unlike this case. In imaging, most of the differential diagnoses present with basal ganglia involvement, without subcortical cysts [2,5].

Treatment and follow-up

The parents received genetic counseling in the absence of any available treatment, and supportive therapy by physical therapy was advised. The patient was asked to follow up two months later.

## Discussion

In this case report, we have described a rare condition with atypical epidemiological traits. MLC is an inherited condition that runs in the family, typically found in groups where consanguinity is widespread, like the Indian Aggarwal population, Jews from Libya, and Turks [3,6].

Though it may be present at birth, macrocephaly usually appears throughout infancy [7]. In our case, the patient was born with macrocephaly, with the head circumference increasing progressively since childhood. This child was having seizure episodes from the age of three years. The disease is thought to present with early-onset seizure.

MRI of the brain can be used as a diagnostic tool since it invariably shows subcortical cysts and diffuse white matter alterations in the anterior frontoparietal area or the temporal region [1]. Later down the road, brain atrophy takes place and white matter swelling decreases. Corpus callosum, internal capsule, brain stem, cerebellar white matter, and basal ganglia remain relatively unaffected.

Our patient presented with classical MRI brain findings of Van der Knaap disease. With the majority of cases (75%) represented by the MLC1 gene on the long arm of chromosome 22q and 20% encoded by HEPACAM on the long arm of chromosome 11 (11q24.2), the disease exhibits autosomal inheritance [8].

The brain, spleen, and leukocytes all contain a plasma membrane protein that is encoded by the MLC1 gene [5,8,9]. The GlialCAM protein is encoded by the HEPACAM gene [5].

When it comes to genetic counseling about the avoidance of customary marriage in some consanguineous marriage communities, the proper identification of the disease based on clinical characterization and MRI findings is crucial [10]. Consanguinity, however, did not play a role in our scenario, nor was the child from the Aggarwal community, which shows an atypical characteristic in the disease's epidemiological trend [10]. This ailment rarely appears in children of India's "non-Aggarwal" population born out of non-consanguineous marriages [10]. There is no specific treatment for the disease, and only supportive care, including physical therapy, can be provided, as was advised in our case.

## Conclusions

In this case study, a patient from a non-Aggarwal society who was born into a non-consanguineous marriage presents with an uncommon form of Van der Knaap disease. While this condition is more commonly associated with communities where consanguinity is prevalent, our case underscores that it can occur in a broader demographic context, demonstrating the importance of considering this diagnosis even in atypical cases. The classical MRI findings were crucial in diagnosing the disease, despite the absence of consanguinity or a high-risk ethnic background. This case emphasizes the need for awareness among clinicians of the diverse presentation patterns of MLC, the significance of genetic counseling, and the current limitations in treatment, which are primarily supportive.
